# Variation of medium osmolalities induces changes in cilia related signalling pathways in primary cultured renal inner medullary collecting duct cells

**DOI:** 10.1186/2046-2530-1-S1-P21

**Published:** 2012-11-16

**Authors:** B Edemir, M Jardzejewski, E Schlatter

**Affiliations:** 1 University Hospital Münster, Germany

## 

Compared to other organs the cells of the renal inner medulla are challenged with an environment of highly variable osmolality. Cellular adaption to changing osmolality is associated with changes in gene expression. To identify novel genes and pathways that are affected by high osmolality we performed microarray experiments using primary cultured rat inner medullary collecting duct cells cultivated at 300, 600, or 900 mosmol/kg for six days. Compared to 300 mosmol/kg more than 2000 genes showed significant changes in expression at 600 or 900 mosmol/kg. Pathway analysis revealed that the WNT/beta-catenin pathway was also affected. Western blot analysis confirmed the down regulation of beta-catenin and of phosphorylated beta-catenin protein. Immunofluorescence analysis indicated massive changes in intracellular localization of beta-catenin. At 300 mosmol/kg beta-catenin was distributed diffusively within the cells. At higher osmolalities beta-catenin was concentrated at the cell to cell contacts and in the nucleus. Similar effects were observed for phosphorylated beta-catenin. We also observed striking changes in cell morphology. While at 300 mosmol/kg the actin filaments were diffusively distributed the cultivation at 600 mosmol/kg was associated with massive reorganisation and enrichment of the actin filaments at the cell to cell contacts. Staining of the microtubules with beta-tubulin clearly showed the primary cilia in cells cultivated at 300 mosmol/kg. Surprisingly the length of the cilia was drastically reduced in cells cultivated at 600 mosmol/kg. The analysis of the underlying physiological mechanisms and the consequences for cilia related signalling at different osmolalities could help to add new aspects to cilia function.

